# High-Pressure Processing—Impacts on the Virulence and Antibiotic Resistance of *Listeria monocytogenes* Isolated from Food and Food Processing Environments

**DOI:** 10.3390/foods12213899

**Published:** 2023-10-24

**Authors:** Patryk Wiśniewski, Wioleta Chajęcka-Wierzchowska, Anna Zadernowska

**Affiliations:** Department of Food Microbiology, Meat Technology and Chemistry, Faculty of Food Science, University of Warmia and Mazury, Plac Cieszyński 1, 10-726 Olsztyn, Poland; wioleta.chajecka@uwm.edu.pl (W.C.-W.); anna.zadernowska@uwm.edu.pl (A.Z.)

**Keywords:** *Listeria monocytogenes*, high-pressure processing, antibiotic resistance, virulence factors, gene expressions, food, food processing environment

## Abstract

High-pressure processing (HPP) is one of the non-thermal methods of food preservation considered to be safe but may cause an increase/decrease in virulence potential and antibiotic resistance. The aim of the present study was to evaluate the survival of *L. monocytogenes* isolates after high-pressure processing (200 and 400 MPa for 5 min) and to determine changes in phenotypic and genotypic antibiotic resistance and virulence after this treatment. The 400 MPa treatment was shown to be effective in reducing pathogens to safe levels; however, the potential for cell recovery during storage was observed. In addition, studies on changes in virulence indicated possibilities related to a decrease in *actA* gene expression, overexpression of the *hly* and *osfX* gene, and an increase in biofilm-forming ability. The studies on changes in antibiotic resistance of isolates showed that all isolates showing initial susceptibility to lincomycin, fosfomycin, trimethoprim/sulfamethoxazole, and tetracycline became resistant to these antibiotics, which was associated with an increase in the values of minimum inhibitory concentrations. An increase in the expression of antibiotic resistance genes (mainly *tetA_1*, *tetA_3*, *tetC*) was also observed (mainly after the application of 200 MPa pressure), which was isolate dependent. However, it is noteworthy that the induced changes were permanent, i.e., they persisted even after the restoration of optimal environmental conditions. The results presented in our work indicate that the stress occurring during HPP can affect both phenotypic and genotypic changes in the virulence and antibiotic resistance potential of pathogens isolated from food and food processing environments. The potential associated with cell recovery and persistence of changes may influence the spread of virulent isolates of pathogens with increased antibiotic resistance in the food and food processing environment.

## 1. Introduction

*Listeria monocytogenes* is one of the most dangerous pathogens posing a threat to public health and the food industry [1]. *L. monocytogenes* is a major concern in the food industry due to its ability to survive in various food preservation conditions. This pathogen can trigger stress response mechanisms, making it challenging to ensure safe and high-quality food. *L. monocytogenes* causes listeriosis, a severe illness, especially dangerous for high-risk individuals such as the elderly, pregnant women, newborns, and immunocompromised individuals. [2]. In 2021, 30 countries reported 2268 confirmed cases of listeriosis in the European Union (EU) and the European Economic Area (EEA). Listeriosis is one of the most serious foodborne diseases with the highest rate of hospitalized cases among all zoonotic diseases under EU surveillance. The occurrence of *L. monocytogenes* (in 2021) varied by food category. The highest values were observed for fish and fishery products, beef or pork meat products, fruits and vegetables, and hard cheeses made from raw or low-heat-treated sheep’s milk (4.6%) [3,4].

*L. monocytogenes* has several different stress response mechanisms related to the alternative sigma factors σB, σC, σH, and σL; the most significant response is σB, which controls over 300 stress and virulence genes [5,6]. The presence of so many different factors that influence the pathogenicity of these bacteria affects the need for constant monitoring of their occurrence in food. Food testing is limited to only determining the presence and abundance of this pathogen, and according to European Commission Regulation 2073/2005 [7], monitoring the presence of *L. monocytogenes* in products where its growth is possible should be conducted before these products enter the market. In such products, *L. monocytogenes* should not be present in 25 g of a sample. Additionally, throughout the product’s shelf life, the number of these bacteria in a sample must not exceed 100 cfu/g. Due to the ability of *L. monocytogenes* to possess cell regeneration of sublethally damaged cells, research should include evaluation of the growth potential of these bacteria during storage [1].

Nowadays, non-thermal methods of food preservation are gaining considerable popularity, among which one of the leading methods is high-pressure processing (HPP). This method is considered to be an innovative strategy to minimize negative impacts on food, and it is gaining much popularity. However, HPP may also affect the activation of stress response mechanisms, including resistance regulation systems, oxidative stress systems, and cell repair systems, among others [8]. Important aspects that have not yet been addressed in the scientific research are issues related to the induction of pathogenicity of *L. monocytogenes* isolates and the expression of antibiotic resistance and virulence genes after a preliminary exposure to HPP [9].

Environmental stress induced by food preservation methods has a significant impact on changes in the antibiotic resistance of microorganisms. Only a few studies have focused on examining the interaction related to the tolerance of isolates to HPP-induced stress and their antibiotic resistance [10,11]. Currently, there are no studies that have focused on the effect of HPP on the potential for increased antibiotic resistance and issues related to the expression of antibiotic resistance and virulence genes of *L. monocytogenes* isolates. Therefore, in response to the need to understand the effect of HPP on the antibiotic resistance and virulence of *L. monocytogenes,* the aims of this study were: (i) to evaluate the survival of *L. monocytogenes* isolates after HPP treatment; (ii) to determine changes in phenotypic and genotypic antibiotic resistance among *L. monocytogenes* after HPP treatment; (iii) to observe changes in phenotypic and genotypic virulence (biofilm and slime production abilities) of isolates among *L. monocytogenes* after HPP treatment.

## 2. Materials and Methods

### 2.1. Characteristics of the Isolates Used in This Study

This study was conducted on isolates from food and food processing environments, from a collection of isolates of the Department of Food Microbiology, Meat Technology and Chemistry of the University of Warmia and Mazury in Olsztyn described in a previous study [12]. A total of six isolates were selected for the study. The critera for selecting the isolates used in this study were to belong to one of two serotypes, i.e., 1/2c or 1/2a, and intermediate resistance or resistance to at least one antibiotic.

Before use, the isolates were stored at −80 °C in microbanks (Biomaxima, Lublin, Poland). Microorganisms were cultured on Tryptic Soy Broth (TSB; Merck, Darmstadt, Germany) at 37 °C for 24 h. For further analysis, 2 mL of each culture was transferred to TSB broth and incubated under the same conditions. The characteristics of the isolates selected for analysis are presented in Table 1.

#### 2.1.1. Phenotypic Antibiotic Resistance Analysis and Determination of the Minimum Inhibitory Concentration (MIC) Value

Previous studies [12] have characterized *L. monocytogenes* isolates for resistance to trimethoprim/sulfamethoxazole (SXT), tetracycline (TET), ciprofloxacin (CIP) and clindamycin (DA). The MIC values were determined only for the antibiotics to which the isolates showed resistance. However, additional analyses were conducted to determine MIC values for all antibiotics. Furthermore, resistance analyses were performed for two more antibiotics, i.e., lincomycin (LIN) and fosfomycin (FOS). As a result, each isolate was characterized for resistance and MIC values for seven antibiotics—SXT, TET, CIP, DA, LIN, and FOS. As criteria for the selection of antibiotics for the study, the possibility of using the antibiotic for the treatment of listeriosis and emerging reports of increased resistance to these antibiotics among *L. monocytogenes* were used.

The testing was performed using the 96-well broth microdilution method as recommended by the European Committee on Antimicrobial Susceptibility Testing (EUCAST 2022) [13] and ISO 20776-1 [14], according to the previously described paper by Gajewska et al. (2022) [15]. Briefly, the prepared 96-well microtiter plates contained 50 μL cation-adjusted Mueller–Hinton broth (Merck, Darmstadt, Germany) with twice the concentration of the antibiotic solution and were inoculated with 50 μL of the culture suspension (0.5° McFarland scale). The final concentration ranges used to determine the MIC for the antibiotics tested were as follows: LIN, 0.125–256 µg/mL; FOS, 0.125–256 µg/mL; SXT, 0.0625–32 µg/mL; TET, 0.125–64 µg/mL; CIP, 0.008–16 µg/mL; DA, 0.0625–32 µg/mL. The 96-well microtiter plates were sealed and incubated at 35 ± 2 °C for 20 h. The MIC value was recorded as the lowest antibiotic concentration at which no visible growth was observed in the wells of the microtiter plates with reference to the guidelines of the Clinical and Laboratory Standards Institute (CLSI) [16]. The results were interpreted as resistant (R), intermediate (I), or susceptible (S) using standard reference values according to EUCAST [13] for *L. monocytogenes*. Additionally, the standards for Staphylococci were used due to the lack of standards for antibiotics not included in EUCAST for *L. monocytogenes*.

#### 2.1.2. Presence of Virulence and Antibiotic Resistance Genes

A previous study [12] analyzed the presence of two LIPI-1 genes, i.e., *hlyA* and *prfA*. In the current study, a further analysis was carried out to evaluate the presence of the following virulence-associated genes included in LIPI-1: *actA*, *plcA*, *plcB*, *mpl*, and *osfX*, which are considered to be the main virulence genes in *L. monocytogenes* Additionally, the presence of eight antibiotic resistance genes encoding resistance to antibiotics used in the treatment of human and animal listeriosis *(lin*, *fosX*, *mprF*, *sulI*, *tetC*, *tetA_1*, *tetA_3*, and *dfrA)* was also examined.

In short, DNA was isolated first. For that, the isolates were streaked on tryptic soy agar (TSA) (Merck, Darmstadt, Germany) from broth cultures (TSB; Merck, Darmstadt, Germany). After a 24-h incubation at 37 °C, genomic DNA isolation using a Genomic Mini DNA Isolation Kit (A&A Biotechnology, Gdańsk, Poland) proceeded according to the manufacturer’s instructions. The obtained genomic DNA samples were stored at −20 °C until further analysis in 200 μL Tris-HCl buffer (10 mM, pH 8.5). The presence of fifteen genes was tested by real-time PCR method in all isolates using specific primers and conditions previously described by Zakrzewski et al. (2023) [17]. Amplifications were performed in 10 µL of the Master Mix reaction containing 5 µL of PowerUp SYBR Green Master Mix (Thermo Fischer Scientific, Waltham, MA, USA), 1 μL of forward and reverse primer (800 nM/μL), and 1 µL of extracted DNA, filled up with ddH2O to a final volume. The PCR run was performed using a RotorGene Q system (Qiagen Inc., Montreal, ON, Canada). All the PCR runs were performed using positive controls and RNase-free water as a negative control. The real-time PCR conditions were as follows: 50 °C for 2 min, 95 °C for 10 min (initial denaturation), 40 cycles of 95 °C for 15 s and 60 s at an annealing temperature specific to each analyzed gene. The specificity of the real-time PCR product was evaluated by constructing the melting curve using a gradual rise between 60 and 95 °C with 0.5 °C increments for 5 s. The sequences of the primers and annealing temperatures are presented in Table S1.

### 2.2. High-Pressure Processing

Each of the tested isolates of *L. monocytogenes* was exposed to stress induced by high-pressure processing. In short, 10 mL of a 24-hour culture of each of the isolates analyzed in TSB (Merck, Darmstadt, Germany) was transferred to low-density polyethylene bottle (Kautex, Bonn, Germany) and subjected to HPP treatments. Two pressure variants (200 MPa and 400 MPa for 5 min) were used in the study. The process parameters were selected based on preliminary studies conducted, the latest literature, and the potential for industrial HPP applications in food preservation. HPP was carried out in a glycol-water solution (1:1, *v*/*v*) using a U4040 high-pressure single chamber (IWC PAN, Warsaw, Poland, Unipress Equipment Division) at a temperature of 20 ± 3 °C. The rate of pressure build-up was 300 MPa/min, while the pressure relief time was <5 s. For each tested isolate, the assays were performed in three independent processes. After the HPP treatments were completed, further steps of this study proceeded. Unpressurized samples were used as a control.

#### 2.2.1. Survival and Recovery Analysis after HPP

The survival of the isolates was checked by the plate count method. For this purpose, a series of ten-fold dilutions was made for each isolate before and after exposure to HPP, and then the number of colony-forming units (CFU/mL) was determined on ALOA agar (agar for *Listeria* according to Ottaviani and Agosti, Merck, Darmstadt, Germany). The number of colonies was counted after 48 h of incubation at 37 °C. The number of CFU/mL was determined immediately after the high-pressure treatment.

If viable cells (not culturable) were not detected by the plate count method (cell count below the detection limit), cells were attempted to be recovered during storage, according to Valdramidis et al. (2015) [18] with our own modifications. The presence or absence of *L. monocytogenes* was monitored immediately after the HPP treatment and during storage (at 1-day intervals until growth was detected, at 30 °C). Briefly, after the HPP treatment, 1 mL of each bacterial culture was transferred to a fresh TSB medium (Merck, Darmstadt, Germany) and incubated at 30 °C to allow cell recovery. Then, the suspension was streaked on TSA medium (Merck, Darmstadt, Germany) after the growth of each isolate was observed (in triplicate). All TSA plates were incubated at 30 °C for 48 h. If growth occurred on the plates, five random colonies were confirmed by culture on ALOA agar (Merck, Darmstadt, Germany) and incubation at 37 °C for 24 h. The entire experiment was repeated on three separate occasions. After recovery, the isolates were cultured on fresh, sterile TSB (Merck, Darmstadt, Germany) and immediately subjected to the next stages of the study.

#### 2.2.2. Change in Phenotypic Antibiotic Resistance Analysis

After applying HPP, the determination of MIC values for six antibiotics proceeded (LIN, FOS, SXT, TET, CIP, and DA). Changes in MIC values were performed directly after stress treatment (200 MPa/5 min), after restoring optimal growth conditions (isolates were cultured in the medium of optimal composition and incubated under optimal conditions), and after the recovery of cells during storage (after exposure to 400 MPa/5 min). MIC values were determined using the methodology described previously in this article (Section 2.1.1. Phenotypic Antibiotic Resistance Analysis and Determination of the Minimum Inhibitory Concentration (MIC) Value). MIC values were read after a 20-h incubation at 35 ± 2 °C. The MIC value was considered to be the lowest antibiotic concentration at which no visible growth was observed in the microtiter plate wells. Values were referenced to CLSI [16] and EUCAST [13] guidelines. Results were interpreted as susceptible, intermediate resistant, or resistant.

#### 2.2.3. Real-Time PCR Analysis

##### RNA Extraction and Reverse Transcription into cDNA

A Total RNA Mini Plus Kit (A&A Biotechnology, Gdynia, Poland) was used to isolate total RNA. After isolation, purification and concentration proceeded according to the manufacturer’s recommendations for the CleanUp RNA Concentrator kit (A&A Biotechnology, Gdynia, Poland). Each RNA sample was subjected to integrity testing by loading 10 µL of RNA into a 1.2% agarose gel in 0.5% TBE buffer and running at 90 V for 1 h. Then, the two bands, 16S and 23S RNA, were visualized by fluorescent staining using a G-BOX F3 gel documentation and analysis system (Syngene, Cambridge, UK). The concentration and purity of the RNA samples were measured by sample absorbance at 260 nm and 280 nm, using a DeNovix DS11 FX spectrophotometer/fluorometer (DeNovix Inc., Wilmington, NC, USA). In the next step, RNA was transcribed into cDNA using the TranScriba kit (A&A Biotechnology, Gdynia, Poland) to synthesize the first strand of cDNA using recombinant MMLV reverse transcriptase, which shows optimal activity at 37–42 °C and low RNAse activity. Random sequence hexameters were used as primers, and the RNA template was protected with a recombinant RNAse inhibitor.

##### Genes Expression

The study evaluated the expression of fifteen genes, i.e., seven virulence-associated genes included in LIPI-1 (*prfA*, *hly*, *actA*, *plcA*, *plcB*, *mpl*, and *osfX*) and eight antibiotic resistance genes (*lin*, *FosX*, *mprF*, *sulI*, *tetC*, *tetA_1*, *tetA_3*, and *dfrA*) in three independently run reactions, fluorometrically using SYBR green with the RotorGene Q system (Qiagen Inc., Montreal, ON, Canada). The qPCR reactions were performed as previously described by Zakrzewski et al. (2023) [17]. Briefly, 5 µL of PowerUp SYBR Green Master Mix (Thermo Fischer Scientific, Waltham, MA, USA), 1 μL of forward and reverse primer (800 nM/μL), and 1 µL of extracted cDNA, filled up with ddH2O to a final volume (10 µL). Cycling conditions were as follows: 50 °C for 2 min, an initial denaturation step of 10 min at 95 °C, followed by 40 cycles of 15 s at 95 °C, and 60 s at an annealing temperature specific to each analyzed gene. The sequences of the primers and annealing temperatures are presented in Table S1. The melting curve was constructed by heating in a slow ramp between 60 and 95 °C in increments of 0.5 °C for 5 s. To determine the expression level of the analyzed genes, a gene encoding 16S rRNA was selected as the housekeeping gene. Samples were tested for differences in gene expression using relative quantification (normalizing gene expression to the housekeeping gene) according to the mathematical model described by Pfaffl (2001) [19]. A threshold was determined by the software for real-time PCR reactions. Expression ratios of 2 or more indicated a significant increase in gene expression, while expression ratios of 0.5 or less indicated a significant decrease in gene expression.

#### 2.2.4. Changes in Biofilm and Slime Production Abilities

After the HPP treatments, changes in biofilm formation ability proceeded using the microtiter plate (MTP) method, previously proposed by Stepanović et al. (2007) [20] and as described by Wiśniewski et al. (2022) [12]. Briefly, the ability to produce a biofilm was tested on 96-well, flat-bottomed, sterile polystyrene plates (Promed^®^). The strength of biofilm formation was determined by measuring absorbance at 570 nm using a spectrophotometric microplate reader Varioscan LUX (Thermo Scientific, San José, CA, USA). The optical density (OD) for each test isolate was examined by taking three replicate measurements at 20 locations in each well. The obtained values were compared with the OD cutoff (ODc) value, which was set as three times the standard deviation above the mean OD of the negative control, which was only BHI broth (Merck, Darmstadt, Germany). The scale proposed by Stepanović et al., 2007 [20] was used to evaluate an isolate’s ability to form biofilm. According to this scale, no biofilm production is an OD value less than or equal to the ODc value, weak biofilm production is ODc < OD ≤ 2x ODc, moderate biofilm production is 2x ODc < OD ≤ 4x ODc, and strong biofilm production is 4x ODc < OD.

Changes in slime production ability were tested using the Congo red agar (CRA) method described previously by [21]. Briefly, the cultures, immediately after HPP, were applied directly to the CRA medium and incubated (37 °C/24 h). After incubation, the plates were stored at room temperature (23 °C) for 48 h. The ability to produce slime was interpreted based on the phenotype of the colonies; black colonies were considered to be positive slime production and dark red and red colonies were considered to be negative slime production.

## 3. Results

### 3.1. Survival Analysis

The survival analysis of the isolates in response to the two pressures was carried out using plate count methods. The application of a pressure of 200 MPa was ineffective in inactivating all six *L. monocytogenes* isolates. However, a pressure of 400 MPa was effective in reducing the number of viable cells below the detection limit (Table 2). The isolates were subjected to recovery after treatment of 400 MPa pressure. The recovery ability of all isolates after 72 h of storage was found.

### 3.2. Changes in Antibiotic Resistance Phenotype and Minimum Inhibitory Concentration (MIC) Value

After applying the HPP treatments, the results showed an increase in MIC values compared to the control samples for LIN, FOS, SXT, TET, CIP, and DA, respectively, in: four isolates (66.7%) (increase value from 0.25–16 to 4–8 µg/mL), five isolates (83.3%) (from 2–>256 to 128–>256 µg/mL), three isolates (50.0%) (from <0.0625–0.125 to 0.0625–0.125 µg/mL), six isolates (100.0%) (from <0.125–0.5 to 1–2 µg/mL), five isolates (83.3%) (from 0.125–0.25 to 0.25 µg/mL), and one isolate (16.7%) (from 0.25–2 to 0.25–1 µg/mL), after applying 200 MPa pressure. Moreover, the values also increased in five isolates (83.3%) (increase value from 0.25–16 to 4–64 µg/mL), four isolates (66.7%) (from 2–>256 to 64–>256 µg/mL), three isolates (50.0%) (from <0.0625–0.125 to 0.0625–0.25 µg/mL), six isolates (100.0%) (from <0.125–0.5 to 1–4 µg/mL), five isolates (83.3%) (from 0.125–0.25 to 1 µg/mL) and two isolates (33.3%) (from 0.25–2 to 0.0625–1.5 µg/mL), after applying 400 MPa pressure.

**Figure 1:**
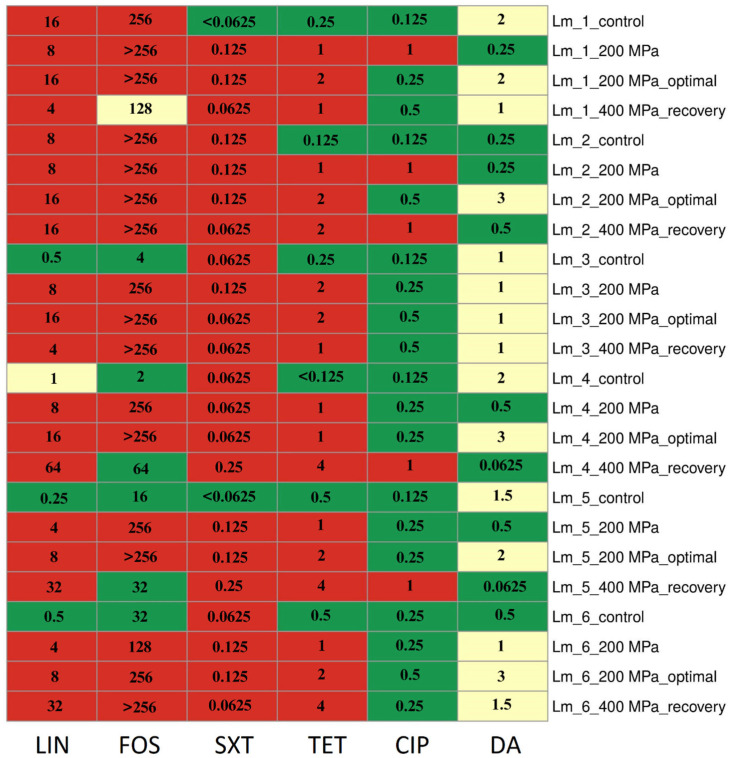
Changes in antibiotic resistance phenotype and MIC (µg/mL) values after HPP treatments. Abbreviation: LIN—lincomycin, FOS—fosfomycin, SXT—trimethoprim/sulfamethoxazole, TET—tetracycline, CIP—ciprofloxacin, DA—clindamycin; control—control sample (before stress treatment); optimal—optimal growth conditions (the isolates were cultured into media with optimal composition and incubated under optimal conditions); recovery—recovered isolates (after exposure to 400 MPa pressure); green—susceptible, yellow—intermediate resistant, red—resistant.

After restoring optimal growth conditions, all isolates for LIN, four isolates (66.7%) for FOS, two isolates (33.3%) for CIP, and five isolates (83.3%) for DA showed an increase in MIC values compared to values after direct application of 200 MPa pressure. Changes in MIC values for antibiotics of isolates are presented in Figure 1.

For all isolates initially exhibiting sensitivity to LIN and FOS (n = 4, 66.7%), SXT (n = 2, 33.3%), and TET (n = 6, 100.0%), there was an increase in MIC values, leading to a shift in resistance classification (to resistant). It is noteworthy that the application of each pressure variant, as well as the restoration of optimal growth conditions, increased the MIC values for TET. These changes were observed following the application of 200 MPa pressure and after the cells’ recovery from the application of 400 MPa pressure. Importantly, these changes persisted even after the restoration of optimal conditions (following the application of 200 MPa pressure) (Figure 1).

### 3.3. Changes in Gene Expression

**Figure 2:**
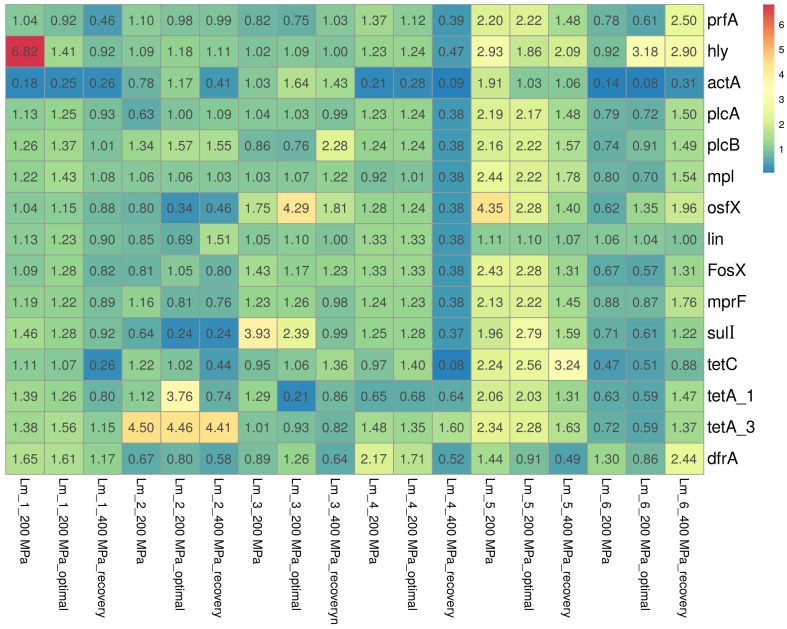
Heatmap of changes in the relative expression levels of the virulence and antibiotic resistance genes in response to HPP treatments. Abbreviation: optimal—optimal growth conditions (the isolates were cultured into media with optimal composition and incubated under optimal conditions); recovery—recovered isolates (after exposure to 400 MPa pressure). Results were visualized using the ClustVis visualizing tool [22].

In the present study, the expression of virulence-associated genes (LIPI-1) and antibiotic resistance genes among *L. monocytogenes* isolates was analyzed. The relative expressions of genes among the tested isolates are shown in Figure 2.

Regarding genes directly associated with virulence (LIPI-1), mainly slight decreases/increases in the relative expression levels of genes were observed, with levels ranging from 0.62 to 1.75, from 0.61 to 1.86, and from 0.88 to 1.96, respectively, for isolates after the application of 200 MPa pressure, after the restoration of optimal conditions (following the application of 200 MPa pressure), and after the application of 400 MPa pressure. Significant underexpression (<0.50) was primarily noted in the *actA* gene, responsible for encoding the major virulence determinant of *L. monocytogenes*, among three isolates (50.0%): Lm_1 (Lm_1_200 MPa (0.18), Lm_1_200 MPa_optimal (0.25), Lm_1_400 MPa_recovery (0.26)); Lm_4 (Lm_4_200 MPa (0.21), Lm_4_200 MPa_optimal (0.28), Lm_4_400 MPa_recovery (0.09)); and Lm_6 (Lm_6_200 MPa (0.14), Lm_6_200 MPa_optimal (0.08), Lm_6_400 MPa_recovery (0.31)). In contrast, overexpression (>2.00) was mainly observed for two genes: *hly* (Lm_1_200 MPa (6.82), Lm_5_200 MPa (2.93), Lm_6_200 MPa_optimal (3.18), Lm_5_400 MPa_recovery (2.90)) and *osfX* (Lm_5_200 MPa (4.35), Lm_3_200 MPa_optimal (4.29), Lm_5_200 MPa_optimal (2.28)).

In general, the genes encoding antibiotic resistance were characterized by similar expression as under optimal conditions; there was only a slight decrease/increase in the expression of genes for which relative expression ranges of 0.63–1.96 (for isolates after applying 200 MPa pressure), 0.51–1.71 (for isolates after restoring optimal conditions after 200 MPa pressure), and 0.52–1.76 (for isolates after applying 400 MPa). However, significant overexpression was observed mainly for three genes encoding tetracycline resistance: *tetA_1* (Lm_5_200 MPa (2.06), Lm_2_200 MPa_optimal (3.76), Lm_5_200 MPa_optimal (2.03))*; tetA_3* (Lm_2_200 MPa (4.50), Lm_5_200 MPa (2.28), Lm_2_200 MPa_optimal (4.46), Lm_5_200 MPa_optimal (2.28), and Lm_2_400 MPa_recovery (4.41)); and *tetC* (Lm_5_200 MPa (2.24), Lm_5_200 MPa_optimal (2.56), Lm_5_400 MPa_recovery (3.24)), with underexpression observed for the *tetC* gene (Lm_6_200 MPa (0.47), Lm_1_400 MPa_recovery (0.26), Lm_2_400 MPa_recovery (0.44), and Lm_4_400 MPa_recovery (0.08)).

In general, it was found that the expression of specific genes depended on the isolate. In the case of isolate Lm_5, there was significant overexpression after application of 200 MPa pressure for six of the seven virulence genes analyzed, i.e., *prfA*, *hly*, *plcA*, *plcB*, *mpl*, and *osfX* (relative expression level 2.16–2.93) and five of the eight antibiotic resistance genes, i.e., *FosX*, *mprF*, *tetC*, *tetA_1*, and *tetA_3* (2.06–4.35), with overexpression persisting even after optimal conditions were restored. Significant underexpression of eleven of the fourteen genes analyzed, i.e., all the LIPI-1 genes (expression ranged from 0.09 to 0.47) and five antibiotic resistance genes, i.e., *osfX*, *lin*, *FosX*, *mprF*, *sulI*, and *tetC* (expression ranged from 0.08 to 0.38) was also observed after applying 400 MPa pressure to the Lm_4 isolate.

### 3.4. Changes in Biofilm and Slime Production Abilities

After applying the HPP treatments, an increase in biofilm production ability was observed in all the analyzed isolates, regardless of the pressure parameter value or their initial biofilm production ability/inability. These changes persisted after the restoration of optimal environmental conditions.

As regards the slime production capacity, the production of slime was only observed for three isolates (50.0%) following the application of a pressure of 200 MPa, with the isolates initially lacking this ability. These changes did not persist once optimal conditions were restored. Changes in biofilm and slime production abilities are summarized in Table 3.

## 4. Discussion

In recent years, the identification and characterization of isolates under environmental stress caused by food processing has been a major research topic [6,23,24]. During food processing, microorganisms experience various environmental conditions unfavorable to their growth, including low and high temperatures, pH, osmotic stress, disinfectants, or high pressures [25]. High pressures as a method for food preservation are gaining in popularity, which is why research has been focusing on assessing their effectiveness. There is also an increase in the number of studies on analyses relating to the cellular responses of microflora that have been intentionally added or can survive the process [21,26]. However, comprehensive studies on the cellular responses of pathogens such as *L. monocytogenes* after high-pressure processing are lacking.

High-pressure processing uses different variants of combinations of mainly three factors: pressure (100–600 MPa), time (from a few to several minutes), and temperature (>23 °C) [27]. The high-pressure process parameters are primarily determined by the product type, based on which the appropriate values of the three HPP parameters are selected. The type of product determines, to a large extent, the possibilities associated with the development of a specific microflora (including pathogenic microflora) [28]. Environmental stress induced by HPP contributes to changes in the microorganism cell structure and the cell genome. Consequently, this contributes to changes in cell pathogenicity, including an increase in virulence and antibiotic resistance, which is linked to an increase/decrease in the expression of the genes responsible for their encoding [26]. The extent of the induced changes is dependent on the HPP parameters [29]. Some changes induced by HPP may not be permanent, and the initial reduction in population may be temporary. Cell regeneration can occur during the storage of food products, which is particularly important for pathogenic microflora [30]. Also, sublethal doses of stress factors during preservation (too low a pressure/too short process duration) may affect the activation of mechanisms responsible for an increase in the antibiotic resistance of isolates, the acquisition of cross-resistance, or the adaptation to environmental stress [31].

*L. monocytogenes* can tolerate a wide variety of adverse environmental conditions occurring during food production [32]. The literature data indicate that the susceptibility of *L. monocytogenes* to HPP is determined by a combination of several factors, i.e., the cell growth phase, individual isolate characteristics, and, primarily, a combination of the conditions under which HPP is carried out [33]. A significant, varying survival rate of different phenotypes and genotypes of *L. monocytogenes* isolates subjected to different HPP variants has been observed [34]. In the current study, two pressure values were selected for HPP (200 and 400 MPa over 5 min) based on the literature data, preliminary research, and the potential for industrial applications. The lower pressure variant did not significantly reduce the population of *L. monocytogenes* in any of the test isolates, which is consistent with the previously conducted studies [26,35,36,37]. The second pressure variant ensured that the *L. monocytogenes* population was reduced below the level of detection by the plate count method. However, cell regeneration was observed after storage at a temperature of 30 °C. In several previous studies, no presence was noted of culturable *L. monocytogenes* cells immediately after 400 MPa pressurization, while after storage (for up to as many as 42 days) in different (even refrigerated) temperatures, cell recovery was noted in both the food and the microbial culture medium [18,38,39]. The literature reports that cell recovery is determined by several factors, i.e., the process duration and temperature, the individual characteristics of the isolate, and the food matrix. [34,35,36,37]. The recovery ability is also explained by the ability of pressurized *L. monocytogenes* cells to transition into a viable but non-culturable (VBNC) state [30]. Such damaged cells can repair the structural changes once optimal environmental conditions are restored during storage at a temperature lower than the optimum growth temperature of these bacteria (especially at the temperatures at which HPP-preserved foods are stored) [40]. The available studies suggest that the ability to adapt to the encountered stress factors may contribute to the ability to survive in one adverse environment while leading to cross-contamination of food products [41].

Microorganisms, particularly pathogens, have various stress response mechanisms that are essential for survival in an adverse food processing environment [42]. *L. monocytogenes* have numerous virulence factors that affect adhesion, binding, and invasion during infection [43]. The genes that are part of Listeria pathogenicity island-1 (LIPI-1) are among the main factors responsible for the pathogenicity of *L. monocytogenes* [44]. The current study found the presence of all the analyzed genes (*plcA*, *mpl*, *actA*, *plcB*, and *osfX*), which are part of LIPI-1, among all the analyzed *L. monocytogenes* isolates (each of the analyzed isolates contained all the LIPI-1 genes). Furthermore, the current study focused on assessing the effect of high-pressure processing on changes in the expression of these genes and changes within the phenotype of biofilm formation and slime production. The study results indicated possibilities associated with the transient acquisition of slime production ability (for a pressure of 200 MPa), which was lost when optimal conditions were restored, and an increase in the biofilm formation strength, independently from the pressure parameters or the initial strength of its formation (even after the restoration of optimal conditions). The literature reports that biofilm formation ability increases under the influence of HPP [35,45]. The current study is the first to show the potential for increasing the strength of biofilm formation, which is a persistent feature.

Our research indicates overexpression (relative expression >2.0) primarily of two genes, *hly*, encoding listeriolysin O (LLO) (a toxin that forms pores, enabling *L. monocytogenes* to escape from host cell phagosomes and undergo intracellular replication [46]) and *osfX* (a virulence factor that dampens the oxidative response of infected macrophages) [47]. Underexpression was mainly observed in the *actA* gene (actin assembly-inducing protein) [44]. The available literature on changes in virulence gene expression in *L. monocytogenes* under the influence of HPP has only focused on a few genes, including overexpression of *plcA* and *hly* genes, as well as suppression of *sigB* and *prfA* gene expression, depending on the isolate [48]. Our research is the first to focus on analyzing the expression of all LIPI-1 genes under the influence of various HPP variants. Our results confirm that the transcription of virulence genes under the influence of HPP is isolate specific, dependent on HPP conditions, and persists after the restoration of optimal conditions and cell cultivability.

Many different classes of antibiotics are used in the treatment of listeriosis in humans, among which gentamicin, amoxicillin, and penicillin are the first-line antibiotics. In the case of pregnant women, treatment involves the use of erythromycin, vancomycin, and trimethoprim/sulfamethoxazole [49]. Listeriosis can also be treated with other antibiotics, including tetracycline, rifampicin, or fluoroquinolones [50]. In our study, we determined resistance and HPP-mediated changes in resistance to six antibiotics, as well as the expression of genes encoding these resistances. It was observed that the HPP treatments increased antibiotic resistance (associated with an increase in MIC values) in isolates initially showing susceptibility to four of them (lincomycin, fosfomycin, trimethoprim/sulfamethoxazole, and tetracycline). Our research also demonstrated that the expression of specific antibiotic resistance genes is likely determined by individual isolate characteristics, the type of treatment, and the time following treatment.

The observed change in the classification of resistance among all the analyzed *L. monocytogenes* isolates for tetracycline as a result of an increase in the MIC value may be related to the significant overexpression of the three analyzed genes encoding resistance to this antibiotic, namely *tetA_1*, *tetA_3,* and *tetC*. There are currently few studies on HPP’s impact on antibiotic resistance changes. There have been reports of increased tetracycline resistance under the influence of this technology among lactic acid bacteria; exposure to HPP affected the expression of the gene encoding resistance to this antibiotic, yet differences were observed between isolates [51,52]. A study conducted by Duru et al. (2020) [10] analyzed the effect of HPP on antibiotic resistance genes *(FosX*, *mprF*, *norB*, and *lin)* also demonstrated differences in the expression of particular antibiotic resistance genes, resulting from individual isolate characteristics and the time after pressure treatment, and also from differences in the amino acid sequence of genes encoding resistance to a specific antibiotic among individual isolates.

The literature reports variability related to individual strains affecting the effectiveness of HPP treatments [53]. Perez-Baltar et al. (2021) [53] examined the survival of two selected strains of *L. monocytogenes* after exposure to HPP in dry-cured ham and during refrigerated storage. The strains, S2 and S7-2, exhibited moderate resistance to HPP (after 450 MPa/10 min, a reduction of 0.8 log CFU/g and after 600 MPa/5 min, reductions of 1.3 and 1.5 log CFU/g, respectively) in low water activity (aw = 0.88) sliced dry-cured ham. Bruschi et al., (2017) [11] used 14 strains of *L. monocytogenes* isolated from food and clinical sources, with different phenotypic and genotypic characteristics. They also observed high intra-strain variability in pressure resistance among the tested strains. In general, all strains were able to survive the 300, 400, and 500 MPa pressure treatments, with loss of viability (in log cycles) ranging from 0.00 to 2.76, from 0.06 to 6.31, and from 0.75 to 7.23, respectively. The application of 500 MPa pressure was enough to reduce the viability of all strains by more than 5 log cycles except for one strain isolated from fermented sausage (reduction <1.00 log CFU/g). The researchers also observed that antibiotic-resistant strains of *L. monocytogenes* showed a higher level of survival after applying 400 MPa pressure [11]. In this study, the application of a pressure of 200 MPa/5 min had no effect on reducing the number of microorganisms; however, a pressure of 400 MPa/5 min had the effect of reducing the number below the detection level for all strains. The increased antibiotic resistance of these strains did not increase their resistance to HPP as in the previous study [11]. Our study also observed marked variability among the isolates in the expression of virulence and antibiotic resistance-related genes and confirmed previous observations, i.e., the way the genes are expressed is a process that may not only significantly vary among different strains of the same bacterial species [54,55]. In addition, studies have reported that gene expression (transcription) was not always correlated with protein synthesis results (translation) for genes involved in biofilm formation in *L. monocytogenes* [56]. Toliopoulos and Giaouris (2023) [55] reported that the strain-related effect of a variation in biofilm gene expression should partly explain the conflicting observations that have sometimes been reported in the literature. In our opinion, strain variability may also influence differences in the expression of other genes encoding virulence and antibiotic resistance among different *L. monocytogenes* isolates. Understanding the links among these characteristics and the differences in antibiotic resistance and gene expression among individual isolates should be a topic for further research consideration, and the results obtained in the current study indicate the need to control changes in antibiotic resistance of pathogens isolated from food under high-pressure processing.

## 5. Conclusions

Food processing stress changes the virulence and antibiotic resistance of pathogens isolated from food. The results of this study enabled verification of the applied HPP treatment parameters in relation to survivability, virulence potential, and antibiotic resistance of *L. monocytogenes* isolates. In addition, the results pointed to the possibilities associated with the recovery of cells of this pathogen during storage and with changes in the expression of genes involved in virulence and antibiotic resistance. The possibility of retaining cellular activity after recovery and after restoration of optimal environmental conditions may directly impact the spread of highly virulent isolates exhibiting increased antibiotic resistance in the food and food processing environments. The results indicate the need for further analysis of the impact of HPP on the virulence and antibiotic resistance of pathogens isolated from food and food processing environments.

## Figures and Tables

**Table 1:** Isolate characterization.

Isolate		Serotype	Isolation Source	Biofilm	Slime Production	LIPI-1	Antibiotic MICs [µg/mL]	Antibiotic Resistance Genes
Lm_1	168	1/2c	Floor drain	Strong	No	*hlyA* *prfA*	DA—2 (I)	*lnuA*
Lm_2	165		Floor drain	Weak			P—1 (R)	*mefA, sulI*
							SXT—0.125 (R)	
Lm_3	177		Production line	Weak			DA—1 (I)	*sulI, sulII*
							SXT—0.064 (R)	
Lm_4	92	1/2a	Juice	Strong			DA—2 (R)	*sulI*
							MEM—0.047 (R)	
							SXT (R)—0.064 (R)	
Lm_5	167		Floor drain	Moderate			DA—1.5 (I)	-
Lm_6	148		Frozen vegetables	No biofilm			CN—0.19 (I)	*aadB, mefA, lnuA, sulII*
							SXT—0.064 (R)	

LIPI-1—Listeria pathogenicity island-1; MIC—minimum inhibitory concentration; I—intermediate; R—resistance; DA—clindamycin, CN—gentamicin, MEM—meropenem, P—penicillin, SXT—trimethoprim/sulfamethoxazole.

**Table 2:** The viable cell counts of *L. monocytogenes* isolates analyzed in this study.

	Control *	200 MPa *	400 MPa *	Recovery after 400 MPa
Lm_1	7.15 ± 0.22 × 10^9^	2.18 ± 0.15 × 10^9^	<10	Yes
Lm_2	2.70 ± 0.20 × 10^9^	1.44 ± 0.10 × 10^9^	<10	
Lm_3	1.91 ± 0.10 × 10^9^	1.64 ± 0.11 × 10^9^	<10	
Lm_4	2.26 ± 0.21 × 10^9^	1.87 ± 0.25 × 10^9^	<10	
Lm_5	2.68 ± 0.10 × 10^9^	1.35 ± 0.12 × 10^9^	<10	
Lm_6	2.08 ± 0.19 × 10^9^	1.77 ± 0.13 × 10^9^	<10	

* Colony forming units (CFU/mL); <10, number of bacteria below the detection limit. Abbreviations: control—control sample (before stress treatment), 200 MPa—directly after exposure to 200 MPa pressure, 400 MPa—directly after exposure to 400 MPa pressure.

**Table 3:** Changes in biofilm and slime production abilities after HPP treatments.

	Biofilm				Slime Production			
	Control	200 MPa	200 MPa_Optimal	400 MPa_Recovery	Control	200 MPa	200 MPa_Optimal	400 MPa_Recovery
Lm_1	Strong	Strong	Strong	Strong	No	Yes	No	No
Lm_2	Weak					Yes		
Lm_3	Weak					Yes		
Lm_4	No					No		
Lm_5	Moderate					No		
Lm_6	No					No		

Abbreviation: control—control sample (before stress treatment), optimal—optimal growth conditions (the isolates were cultured into media with optimal composition and incubated under optimal conditions), recovery—recovered isolates (after exposure to 400 MPa pressure).

## Data Availability

The data presented in this study are available in this article and Supplementary Materials.

## Supplementary Materials

The following supporting information can be downloaded at: https://www.mdpi.com/article/10.3390/foods12213899/s1, Table S1: Primers used in the study.
