# Supplementary material for: High-Pressure Processing—Impacts on the Virulence and Antibiotic Resistance of Listeria monocytogenes Isolated from Food and Food Processing Environments
Source: Foods. 2023 Oct 24;12(21):3899. doi: 10.3390/foods12213899 (PMC10650155; doi:10.3390/foods12213899)
Supplement: Supplementary file 1 [file foods-12-03899-s001.zip › foods-2653685-supplementary.pdf]

Table S1. Primers used in study.

	Primer	Sequence 5'-3'	Annealing temperaturę [°C]	Reference
**Virulence associated genes (LIPI-1)**	rt-actA-F	ACAACGGTGAGCAAACAGGA	59.0	[1]
	rt-actA-R	CGCTCCACTTGTAGAGTTGGT		
	rt-hly-F	TGCCAGGTAACGCGAGAAAT	58.5	
	rt-hly-R	TGGTGCCCCAGATGGAGATA		
	rt-mpl-F	ATCAAGGTCCACGTCACACC	58.6	
	rt-mpl-R	TCCTAAGCTGTCCTTGCTGC		
	rt-mprF-F	GGAAGCAGCGATTGAAGAGG	59.4	this study
	rt-mprF-R	GGACGTCGACAAAACCTTCC		
	rt-osfX-F	TGTAATCGCGCTATGTTCCGT	59.8	[1]
	rt-osfX-R	ACTCAATCTCCTTTGCATCGC		
	rt-plcA-F	CCCATTAGGCGGGAAAGCAT	59.6	
	rt-plcA-R	TCAGGTAGAGCGGACATCCA		
	rt-plcB-F	AAAAGGGCGAAAGCGGACTA	57.9	
	rt-plcB-R	ACCTGCCAAAGTTTGCTGTG		
	rt-prfA-F	TTAGCGAGAACGGGACCATC	58.2	
	rt-prfA-R	TAACGTATGCGGTAGCCTGC		
**Antibiotic resistance genes**	rt-dfrA-F	AAGCTGTTTTAGCCCTTGCTG	59.8	
	rt-dfrA-R	GGAAACGCAGTATCAGCCTCA		
	rt-lin-F	AGCGTTCAAACCAAGCAAGT	56.9	
	rt-lin-R	GGAGCATACTGAGCAGCAAC		
	rt-fosX-F	GTGGATGAATATATTGAGCGGATT	56.9	
	rt-fosX-R	CTTCGACTCTGGGACGTTCT		
	rt-sul-F	ATGGTGGAAGATGGAGCAGC	59.0	
	rt-sul-R	TCGGGTAATTTCGTCCTGACA		
	rt-tetA_1-F	GGGCGCTTTGGGTACTCTTT	59.7	
	rt-tetA_1-R	TCCGCTCCTCTTGAGGTGTA		
	rt-tetA_3-F	CGACTGCCAATGCGCTAATC	58.1	
	rt-tetA_3-R	CCAATTAGCACGCCCGAAAC		
	rt-tetC-F	AAAAGAAGCGGACTCGAGCA	58.6	
	rt-tetC-R	AGTGCCGGTTGGGTAATGTT		

[1] Zakrzewski, A.; Gajewska, J.; Chajęcka-Wierzchowska, W.; Zadernowska, A. Effect of sous-vide processing of fish on the virulence and antibiotic resistance of Listeria monocytogenes. NFS J. 2023, 31, 155–161. https://doi.org/10.1016/j.nfs.2023.05.003.
